# Isolation of an Antiaromatic 9‐Hydroxy Fluorenyl Cation

**DOI:** 10.1002/chem.202100786

**Published:** 2021-05-06

**Authors:** Daniel Duvinage, Stefan Mebs, Jens Beckmann

**Affiliations:** ^1^ Institut für Anorganische Chemie und Kristallographie Universität Bremen Leobener Straße 7 28359 Bremen Germany; ^2^ Institut für Experimentalphysik Freie Universität Berlin Arnimallee 14 14195 Berlin Germany

**Keywords:** acylium ion, antiaromaticity, carbocation, electrophilic substitution, fluorenyl ion

## Abstract

Fluorenyl cations are textbook examples of 4π electron antiaromatic five‐membered ring systems. So far, they were reported only as short‐lived intermediates generated under superacidic conditions or by flash photolysis. Attempts to prepare a *m*‐terphenyl acylium cation by fluoride abstraction from a benzoyl fluoride gave rise to an isolable 9‐hydroxy fluorenyl cation that formed by an intramolecular electrophilic attack at a flanking mesityl group prior to a 1,2‐methyl shift and proton transfer to oxygen.

## Graphical abstract

**Surprisingly stable after all**: Attempts to prepare a *m*‐terphenylacylium ion by fluoride abstraction from a benzoyl fluoride, gave rise to a 4π‐electron antiaromatic 9‐hydroxy fluorenyl cation that formed via an intramolecular electrophilic attack at a flanking mesityl group prior to a 1,2‐methyl shift and proton transfer to oxygen.

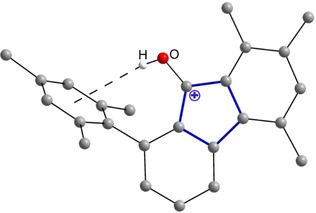

Carbocations are key intermediates in numerous organic reactions. Groundbreaking work by Olah in the 1970s demonstrated that many of these transient carbocations can be detected at low temperatures under superacidic conditions. However, most attempts to isolate these highly reactive species were impaired by decomposition at the time.^[1]^ With the introduction of new powerful Lewis acids and weakly coordinating anions, the situation gradually changed and in recent years the isolation and full characterization of many carbocations was achieved including a non‐classical 2‐norbornyl cation,^[2]^ the benzenium ion,^[3]^ hexahalobenzene radical cations,^[4]^ the hexamethylbenzene dication^[5]^ and cationic ring systems.^[6]^

Acylium ions play a significant role as intermediates in Friedel‐Crafts reactions. In the solid state most acylium ions form ion pairs with their counterions.^[7]^ This observation prompted us to attempt the synthesis of a kinetically‐stabilized *m*‐terphenylacylium ion, in which two flanking mesityl groups were supposed to prevent the coordination of the counterion. Surprisingly, the desired acylium ion immediately underwent an intramolecular Friedel‐Crafts reaction with one of the mesityl groups giving rise to an unexpectably stable 9‐hydroxy fluorenyl cation that was isolated and fully characterized in this work.

Fluorenyl cations comprise a central antiaromatic 4π electron five‐membered ring with two annelated benzene rings (Scheme 1). Motivated by the debate^[8]^ that the antiaromaticity^[9]^ might be compensated by the two benzene rings, serveral attempts were made to spectroscopically characterize or even isolate fluorenyl cations. In 1980, Olah *et al*. obtained several 9‐fluorenyl cations under superacidic conditions including the 9‐hydroxy fluorenyl cation that was generated by protonation of 9‐fluorenone with HSO_3_F/SbF_5_ (“magic acid”) in SO_2_ClF solution at −78 °C.^[10]^ All attempts to isolate these species failed and provided only ill‐defined polymeric materials instead. As the bulk synthesis of 9‐fluorenyl cations deemed impossible, all latter work focused on the photolysis of appropriate precursors, such as 9‐fluorenol or 9‐diazofluorene, in solution and amorphous water ice / neon matrices, respectively.^[11]^

**Scheme 1:**
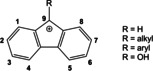
9‐Fluorenyl cations.

We have now found that fluoride abstraction from the benzoyl fluoride 2,6‐Mes_2_C_6_H_3_C(O)F (**1**)^[12]^ with an excess AlCl_3_ provided 9‐hydroxy‐1‐mesityl‐5,7,8‐trimethyl fluorenylium tetrachloroaluminate (**2**) as deep brown crystals in 91 % yield (Figure 1). The fluorenyl cation **2** is stable in chlorinated NMR solvents for a short period of time, but slowly degrades over the course of one day. In donor solvents (e. g. THF, Et_2_O) it turns immediately yellow. Even as a solid under inert conditions, it slowly degrades to become a yellow powder over the course of a few weeks at room temperature. Controlled deprotonation of **2** with NaOH afforded the related 9‐fluorenone **3** as bright yellow crystals in quantitiatve yield. The identity of **1–3** was inferred by the full assignment of the ^1^H and ^13^C NMR spectra and confirmed by X‐ray structure determination (Figure 1).^[13]^ In solution, **1** is characterized by its ^19^F NMR chemical shift (CDCl_3_) of δ=52.2 ppm. It reveals a doublet in the ^13^C NMR spectrum for the *ipso* carbon atom at δ=157.7 ppm with a coupling constant of ^1^ *J*(^13^C−^19^F)=357.2 Hz. Upon fluoride abstraction, the ^13^C spectrum (CD_2_Cl_2_) of **2** shows a more strongly deshielded singlet at δ=200.6 ppm for the *ipso* carbon atom. Furthermore, a new broad signal in the ^1^H NMR spectrum (CD_2_Cl_2_) became visible at δ=9.71 ppm, which was assigned to the hydroxyl group in 9‐position being involved in hydrogen bonding with the π‐system of the mesityl ring. This value is significantly less deshielded than that observed for the parent 9‐hydroxy fluorenylium ion (δ=12.75 ppm), reported by Olah *et al*.^[10]^ According to their work, the relative deshielding of this proton is a measure for the charge density residing on the oxygen atom. Following along this argument, **2** possess a rather low positive charge density there.^[14]^ In **3**, the ^13^C NMR (CDCl_3_) resonance shows a nearly unchanged singlet at δ=195.3 ppm. A UV/vis spectrum of **2** in CH_2_Cl_2_ (50 μM) shows a broad absorption maximum at λ_max_=421 nm, which is also present in **3**. In addition, **3** shows also two absorptions at λ_max_=333 and 346 nm and shows a green‐yellow luminescence with an excitation maximum of λ_max_=320 nm and an emission maximum at λ_max_=512 nm.

**Figure 1:**
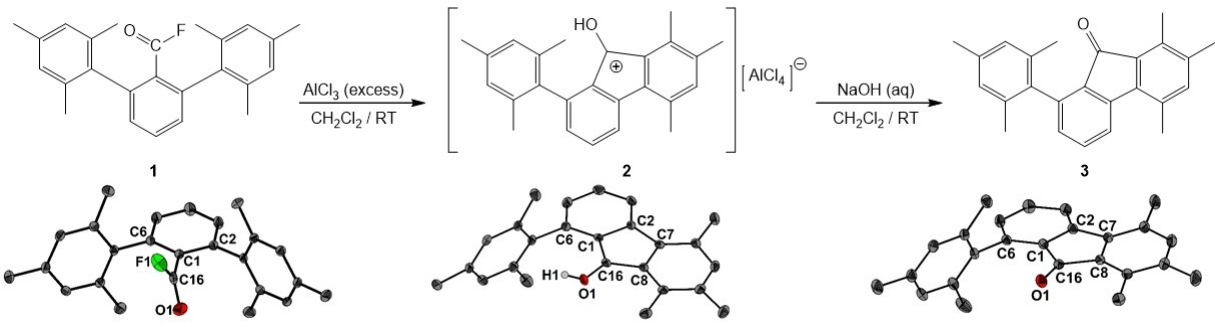
Fluoride abstraction from **1** afforded the 9‐hydroxy fluorenylium tetrachloroaluminate **2**. Base hydrolysis of **2** gave the 9‐fluorenone **3**. Molecular structures of **1**, **2** (counter ion omitted for clarity) and **3** showing 50 % probability ellipsoids and the atom numbering scheme. Selected bond lengths [Å] of **1**: C1‐C2 1.4026(16), C1‐C16 1.4894(17), C2‐C7 1.4983(16), C7‐C8 1.4042(17), O1‐C16 1.2009(17), F1‐C16 1.3308(16). Selected bond lengths [Å] of **2**: C1‐C2 1.416(3), C1‐C16 1.442(3), C2‐C7 1.487(3), C7‐C8 1.418(3), C8‐C16 1.444(3), O1‐C16 1.287(2). Selected bond lengths [Å] of **3**: C1‐C2 1.406(3), C1‐C16 1.496(3), C2‐C7 1.484(3), C7‐C8 1.414(3), C8‐C16 1.501(3), O1‐C16 1.209(3).

The molecular structures^[13]^ of **2** and **3** reveal the presence of fluorene scaffolds and indicate that a methyl group migration had taken place. In both five‐membered ring structures, the C2‐C7, C1‐C16 and C8‐C16 bond lengths are considerably longer than C1‐C2 and C7‐C8, which are shared with the annelated benzene rings. In the fluorenyl cation **2**, the C−O bond length (1.287(2) Å) is significantly longer than in the fluorenone **3** (1.209(3) Å). Both values closely resemble those of protonated cyclopentanone (1.266(3) Å) and cyclopentanone (1.211(2) Å).^[15]^ In **2**, the hydroxyl group points towards the π‐electrons of the remaining mesityl group. In an effort to shed some light on the mechanism that led to the formation of **2**, DFT calculations were carried out at the B3PW91/6‐311+G* level of theory (Figure 2). The relative energy of the assumed initial product of the fluoride abstraction, namely the *m*‐terphenylacylium ion, [2,6‐Mes_2_C_6_H_3_CO]^+^ (**A**) was arbitrarily set to zero. The acylium ion **A** undergoes an electrophilic attack at the *ortho*‐position of one flanking mesityl group. The product of this attack, the arenium ion **C** is by 30 kJ mol^−1^ energetically less favoured. Next, a 1,2‐methyl shift takes place, which gave the arenium ion **E**, which is by 36.8 kJ mol^−1^ energetically even less favoured than the acylium ion **A**. Only the last step is by −114 kJ mol^−1^ energetically favoured. It entails a proton transfer from **E** to the fluorenyl cation **G** (the cation of **2**). This last step involves the largest activation barrier, as the transition state **F^#^** is 154.2 kJ mol^−1^ higher in energy than **A**. In a future study it should be taken into consideration if the proton transfer might as well be mediated by the [AlCl_4_]^−^ ion.

**Figure 2:**
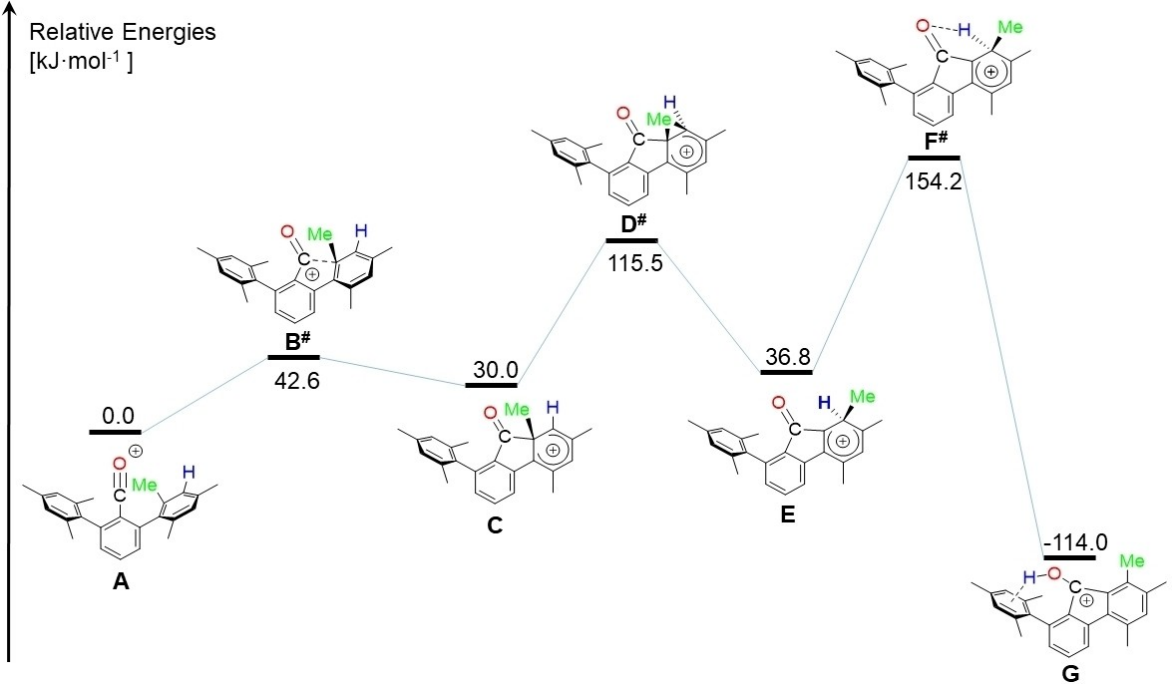
Suggested mechanism of the rearrangement the presumed initial acylium ion **A** into the 9‐hydroxy fluorenyl cation **G** (the cation of **2**). Transition states are marked by superscripted #.

In order to qualitatively monitor the processes of bond formation and rapture along the proposed reaction coordinate, the Atoms‐In–Molecules (AIM)^[16]^ and non‐covalent interactions index (NCI)^[17]^ methods were applied to the DFT models **A**–**G** (Figure 3 and Figures S1–S3). The former provides a molecular graph exceeding the Lewis picture of chemical bonding, whereas the latter provides contact patches even for very weak interactions which not necessarily form a bond critical point (bcp) in AIM. The AIM graph of the transition state **B^#^** (electrophilic attack) closely resembles that of the intermediate **C** in that the CO‐fragment is considerably bent and a C(O)⋅⋅⋅C_ortho_ bcp is already formed despite the large C⋅⋅⋅C distance of 1.942 Å (Figure 3a). The O atom, now closer to the mesityl part on the opposite side, forms a weak O⋅⋅⋅H−C hydrogen bond, which is also visible in the NCI together with even weaker H⋅⋅⋅H contacts. In the transition state of the subsequent 1,2‐methyl shift (**D^#^**) the methyl C atom is still somewhat closer to the *ortho*‐position (1.873 Å) than to the *meta*‐position (1.880 Å). Accordingly, the AIM bond path, which approaches the C_ortho_‐C_meta_ bcp, bends away and finally connects the methyl C atom with the *ortho* C atom, resulting in a quasi T‐shaped bonding scenario (Figure 3b). It might be stated, however, that **D^#^** is closer to the educt (intermediate **C**) than to the product (intermediate **E**). In the latter a second O⋅⋅⋅H−C hydrogen bond is then established. The energy demanding proton transfer (transition state **F^#^**) shows that the proton in *meta*‐position is still topologically connected to the mesityl ring despite a long C−H distance of 1.381 Å (Figure 3c). The out‐of‐plane bending (C_ortho’_‐C_meta_‐C_methyl_ angle; C_ortho’_ is the *ortho* C atom on the opposite side of the mesityl ring) of the methyl group is reduced from 128° (intermediate **C**) to 152° (**F^#^**), which is half way to the value of 178° found in the final compound **G**. It is noted that the experimentally observed intramolecular hydrogen bridge is also present in the optimized structure of **G**, which suggests that it possesses a stabilizing effect to some extent. The NCI proves that non‐covalent interactions are of minor importance for the reaction steps. Notably, transition states which are closer to the product are lower in energy than those which are closer to the starting compound in the current study.

**Figure 3:**
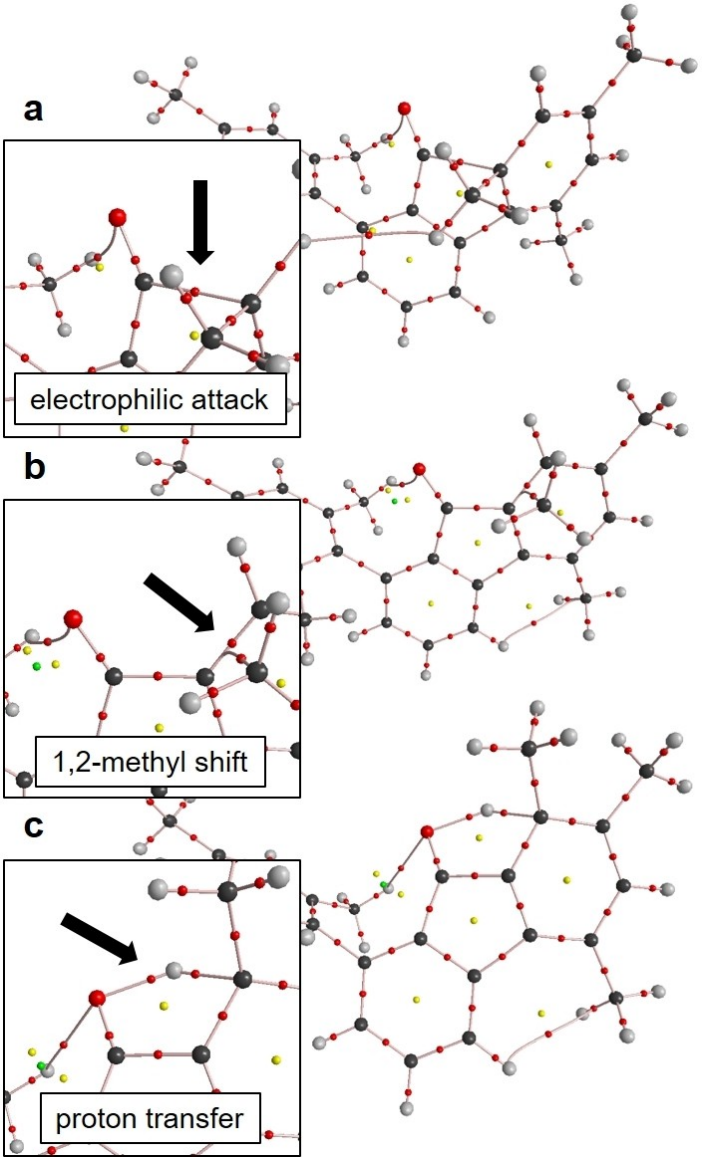
AIM bond topology of the transition states **B^#^**, **D^#^**, and **F^#^** ^.^ Color code atoms: dark grey – C, light gray – H, ref – O. Color code AIM critical points: red – bond critical point (bcp), yellow – ring critical point (rcp), green – cage critical point (ccp). Regions of electrophilic attack, 1,2‐methyl shift, and proton transfer are magnified and highlighted by black arrows.

The cyclopentadienyl cation as well as the central five‐membered ring in the fluorenyl cation formally possess 4π electrons and according to the Hückel rule are antiaromatic, but it is still unclear what influence the two annelated aromatic benzene rings and the hydroxyl group in 9‐position pose on the central five‐membered ring.^[8]^ In an effort to address this question, we calculated nuclear‐independent chemical shifts (NICS) for a number of aromatic and antiaromatic parents compounds (Figure 4).^[18]^ The NICS(0)_iso_ values of benzene and the cyclopentadienyl cation, determined at the ring critical points (rcp) are −8.08 and −13.14, whereas the NICS(1)_iso_ values, referring to points perpendicular to the ring, 1 Å above the rcps are −10.21 and −10.45.^[19]^ Compared to these clearly aromatic benchmarks, the anti‐aromatic cyclopentadienyl cation shows the largest deviation with NICS(0)_iso_ and NICS(1)_iso_ values of 88.96 and 67.45. Introduction of the hydroxyl group in 9‐position dramatically reduces these values to 25.89 and 16.25. Going from the cyclopentadienyl cation to the fluorenyl cation has about the same effect as the values of the 5‐membered ring decrease to 30.03 and 20.11. In turn, the NICS(0)_iso_ and NICS(1)_iso_ values of the two annelated aromatic benzene rings increase to 10.85 and 5.55 when compared to benzene. Both effects are accumulated in the 9‐hydroxyfluorenyl cation with NICS(0)_iso_ and NICS(1)_iso_ values of 18.70 and 11.75 for the central five‐membered ring. These values are very close to those calculated for **G** (Table S2). Thus, the two annelated aromatic benzene rings and the 9‐hydroxy group outweigh the antiaromatic character, which provides a reasonable explanation as to why it was possible to isolate **2**.

**Figure 4:**
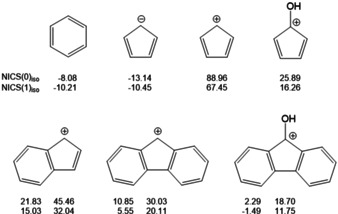
NICS values of aromatic and antiaromatic parent compounds.

In summary, attempts to prepare a kinetically stabilized *m*‐terphenyl acylium ion by fluoride abstraction from the benzoyl fluoride **1** gave the 9‐hydroxy‐1‐mesityl‐5,7,8‐trimethyl fluorenyl cation **2** instead. The rearrangement was rationalized by an intramolecular electrophilic attack (Friedel Crafts reaction) of the initially formed *m*‐terphenylacylium ion, [2,6‐Mes_2_C_6_H_3_CO]^+^ at a flanking mesityl group, prior to a 1,2‐methyl shift and a proton transfer to oxygen. This rearrangement is strongly reminiscent of the reaction between 2,6‐(4‐*t*‐BuC_6_H_4_)C_6_H_3_Li with Cl_2_BH⋅SMe_2_, which gave 9‐bora‐fluorene rather than the expected bis(*m*‐terphenyl)borane.^[20]^ It also resembles our previous attempts to prepare stable bis(*m*‐terphenyl)phosphenium ions by fluoride abstraction from (2,6‐Mes_2_C_6_H_3_)_2_PF and [2,6‐(Me_5_C_6_)_2_C_6_H_3_]_2_PF, which gave a (protonated) 9‐phospha‐fluorene^[21]^ and isomeric 9‐phospha‐fluorenium ions instead.^[22]^ The latter two reactions also involved methyl group migrations. The 9‐hydroxy fluorenyl cation **2** comprises a central five‐membered ring with formally 4π electron, thus fulfilling the Hückel rule for antiaromaticity. The calculation of NICS values suggest that the antiaromatic character is compensated by the accumulative effect of the two annelated benzene rings and the hydroxyl group in 9‐position.

## Conflict of interest

The authors declare no conflict of interest.

## Supporting information

As a service to our authors and readers, this journal provides supporting information supplied by the authors. Such materials are peer reviewed and may be re‐organized for online delivery, but are not copy‐edited or typeset. Technical support issues arising from supporting information (other than missing files) should be addressed to the authors.

Supplementary
