# Supplementary material for: Isolation of an Antiaromatic 9‐Hydroxy Fluorenyl Cation
Source: Chemistry. 2021 May 6;27(31):8105–9. doi: 10.1002/chem.202100786 (PMC8252458; doi:10.1002/chem.202100786)
Supplement: Supplementary file 1 — Supplementary [file CHEM-27-8105-s001.pdf]

Chemistry–A European Journal

Supporting Information

Isolation of an Antiaromatic 9-Hydroxy Fluorenyl Cation

Daniel Duvinage, Stefan Mebs,* and Jens Beckmann*

Table of Contents

Experimental procedures 2

General information 2

Synthesis and characterization of $Mes_2C_6H_3C(O)OH$ 3

Synthesis and characterization of $Mes_2C_6H_3C(O)Cl$ 3

Synthesis and characterization of **1** 3

Synthesis and characterization of **2** 7

Synthesis and characterization of **3** 11

X-Ray diffraction studies 17

Computational data 19

Addtional References 20

Experimental procedures

General information

Unless otherwise stated, all reactions and manipulations were performed under inert atmosphere (argon) using anhydrous solvents. Reagents used in this work including CO_2, $SOCl_2$, pyridine, $AlCl_3$ and CsF were obtained commercially and were used as received. The reagent 2,6-$Mes_2C_6H_3Li$ (Mes = 2,4,6-$Me_3C_6H_2$) was prepared following the published procedure.[S1] Anhydrous dichloromethane, hexane, tetrahydrofurane and toluene were collected from an SPS800 mBraun solvent purification system and stored over 4 Å molecular sieves. Et_2O was dried by refluxing it over Na/benzophenone under argon atmosphere. Deuterated solvents were degassed and dried over 4 Å molecular sieves under argon.

Unless otherwise noted, NMR spectra were recorded at room temperature on a Bruker Avance 600 MHz spectrometer. ^1H, $^{13}C\{^1H\}$ and ^{19}F NMR spectra are reported on the δ scale (ppm) and are referenced against $SiMe_4$ respectively. ^1H and $^{13}C\{^1H\}$ chemical shifts are reported relative to the residual peak of the solvent ($CDHCl_2$ 5.32 ppm for CD_2Cl_2) in the ^1H NMR spectra, and to the peak of the deuterated solvent (CD_2Cl_2 53.84 ppm) in the $^{13}C\{^1H\}$ NMR spectra.[8] The assignment of the ^1H and $^{13}C\{^1H\}$ resonance signals was made in accordance with the COSY, HSQC and HMBC spectra. The labelling schemes are attached to the ^1H and ^{13}C spectra.

The ESI HRMS spectra were measured on a Bruker Impact II spectrometer. Acetonitrile or dichloromethane/acetonitrile solutions ($c = 1\cdot10^{-5}$ mol·L^{-1}) were injected directly into the spectrometer at a flow rate of 3 µL·min^{-1}. Nitrogen was used both as a drying gas and for nebulization with flow rates of approximately 5 L·min^{-1} and a pressure of 5 psi. Pressure in the mass analyser region was usually about $1\cdot10^{-5}$ mbar. Spectra were collected for 1 min and averaged. The nozzle-skimmer voltage was adjusted individually for each measurement.

The UV-Vis spectra were recorded on a Varian Cary 50 Scan UV-Visible Spectrophotometer.

The Fluorescence spectra were recorded on a Jasco Spectrofluorometer FP-8300. The spectral correction was done with an integrated calibrated WI lamp.

Synthesis of $Mes_2C_6H_3C(O)OH$ according to a modified literature procedure.[S2] CO_2 was bubbled through a solution of 2,6-$Mes_2C_6H_3Li$ (3.20 g, 10.0 mmol, 1.00 eq.) in Et_2O (50 mL) at 0 °C. After 1 hour to the suspension was added aqueous HCl (50.0 mL, 3 M) and the reaction was worked up aqueous. The organic phase was separated and the solvent was removed under reduced pressure. The remaining colorless solid is washed with *n*-hexane (3 x 10 mL) and dried under reduced pressure to yield the title compound as colorless solid (3.19 g, 89 %). The analytical data are in accordance with the literature.[S2]

Synthesis and characterization of $Mes_2C_6H_3C(O)Cl$. $Mes_2C_6H_3C(O)Cl$ was prepared according to the literature.[S3] To a stirred suspension of $Mes_2C_6H_3C(O)OH$ (1.79 g, 5.00 mmol, 1.00 eq.) in CH_2Cl_2 (20 mL) thionyl chloride (1.10 mL, 15.0 mmol, 3.00 eq.) was added and after this pyridine (0.10 mL, 1.20 mmol, 0.24 eq.) was added dropwise which starts an evolution of SO_2 and HCl. The solution is stirred for an additional 18 h after which the solvent is removed in vacuum to remove excess thionyl chloride and the residue is dissolved in CH_2Cl_2 (20 mL). The solution is washed with water (3 x 50 mL) and dried over sodium sulphate. The volatiles are removed under reduced pressure to yield the target compound as colorless crystalline solid (1.76 g, 94%). The analytical data are in accordance with the literature.[S3]

Synthesis and characterization of 1. 2,6-$Mes_2C_6H_3C(O)Cl$ (0.50 g, 1.32 mmol, 1.00 eq.) is suspended with CsF (0.50 g, 3.2 mmol, 2.20 eq.) in MeCN (10 mL) and warmed to 80 °C for 48 hours. Afterwards the reaction diluted with CH_2Cl_2 (40 mL) and washed with distilled water (3 x 20 mL). The solvent is removed under reduced pressure and the colorless residue is washed with *n*-hexane (3 x 10 mL) to afford $Mes_2C_6H_3C(O)F$ as colorless solid (0.38 g, 80 %; Mp. 255°C).

^{1}H NMR (600 MHz, $CDCl_3$): δ = 7.65 (t, $^3J(^1H-^1H)$ = 7.66 Hz, 1H, H4), 7.22 (d, $^3J(^1H-^1H)$ = 7.66 Hz, 2H, H3 and H5), 6.94 (s, 4H, H9 and H11), 2.33 (s, 6H, H14), 2.04 (s, 12H, H13 and H15) ppm. **$^{13}C\{^1H\}$ NMR (151 MHz, $CDCl_3$)**: δ = 157.70 (d, $^1J(^{13}C-^{19}F)$ = 357.18 Hz, C16), 140.80 (s, C2 and C6), 137.75 (s, C10), 136.11 (s, C8 and C12) 135.68 (s, C7), 131.96 (s, C4), 129.25 (d, $^2J(^{13}C-^{19}F)$ = 53.70 Hz, C1), 128.98 (s, C3 and C5), 128.26 (s, C9 and C11), 21.26 (s, C14), 20.43 (s, C13 and C15) ppm. **$^{19}F\{^1H\}$ NMR ($CDCl_3$, 565 MHz)**: δ = 52.17 ppm. **HRMS ESI (m/z):** $[M+Na]^+$ calculated. for $C_{25}H_{25}FNaO$, 383.17816; found, 383.17823.

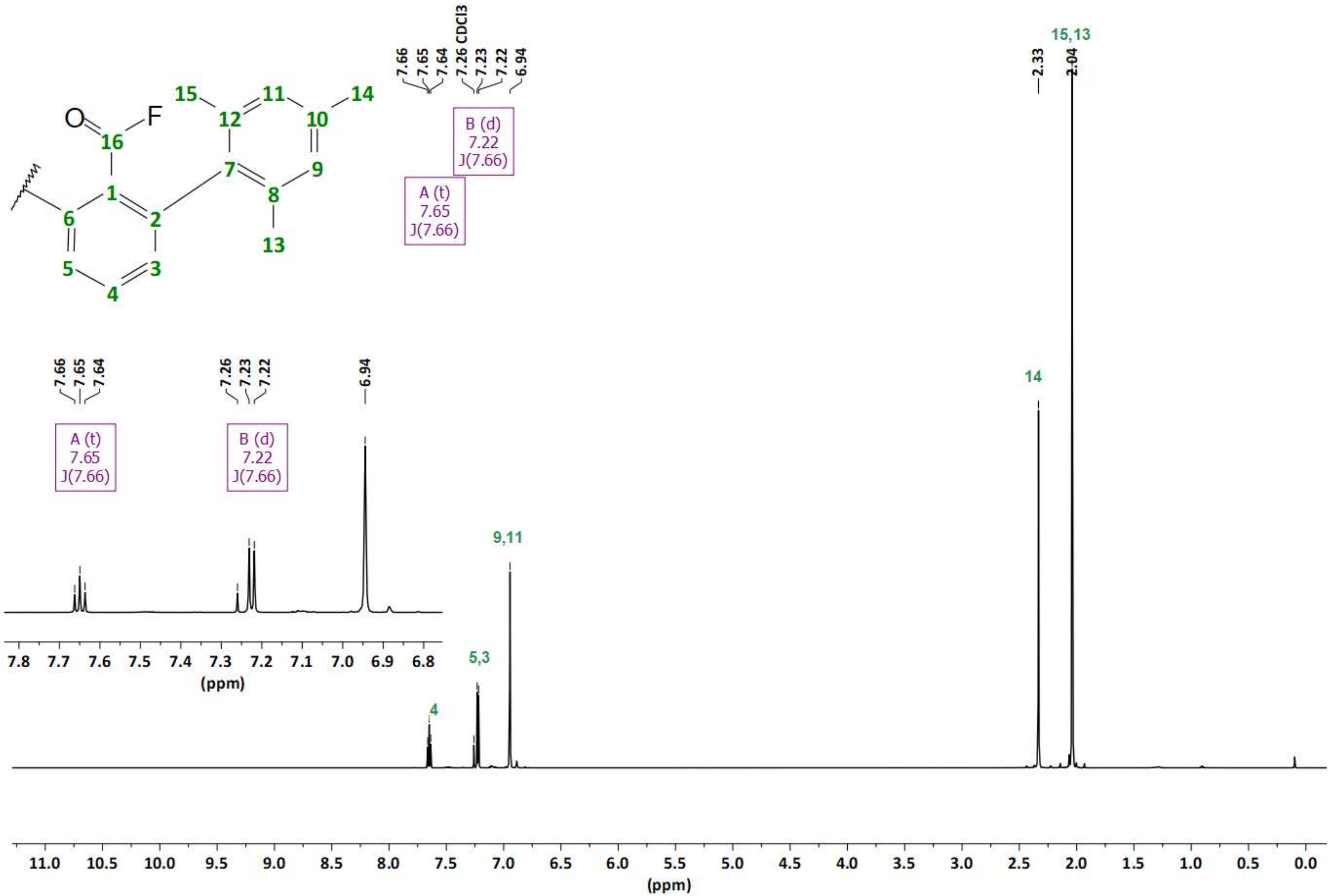

Figure S1. ^1H NMR ($CDCl_3$, 600 MHz) spectrum of 2,6-$Mes_2C_6H_3C(O)F$.

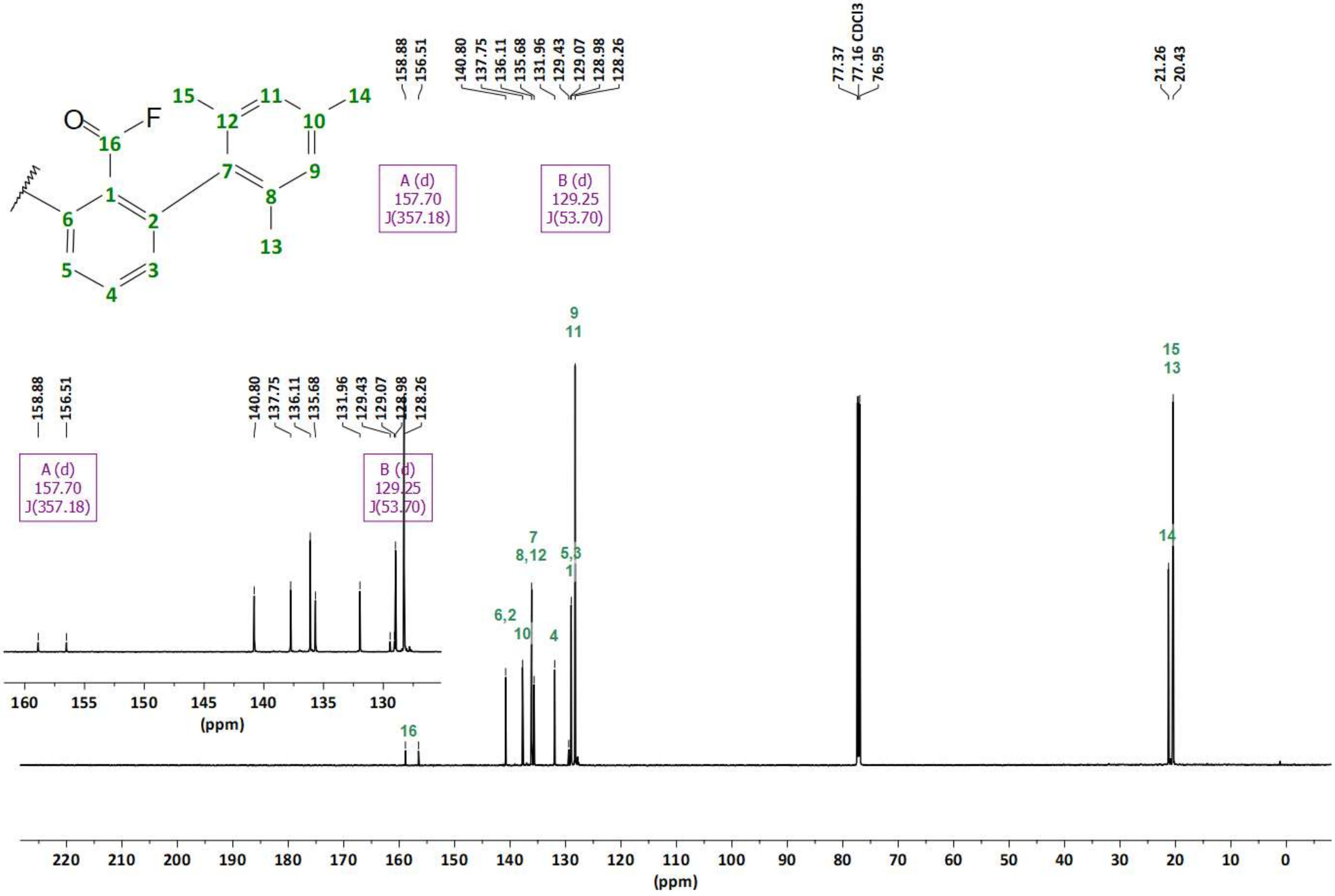

Figure S2. $^{13}C\{^{1}H\}$ NMR ($CDCl_3$, 151 MHz) spectrum of 2,6-$Mes_2C_6H_3C(O)F$.

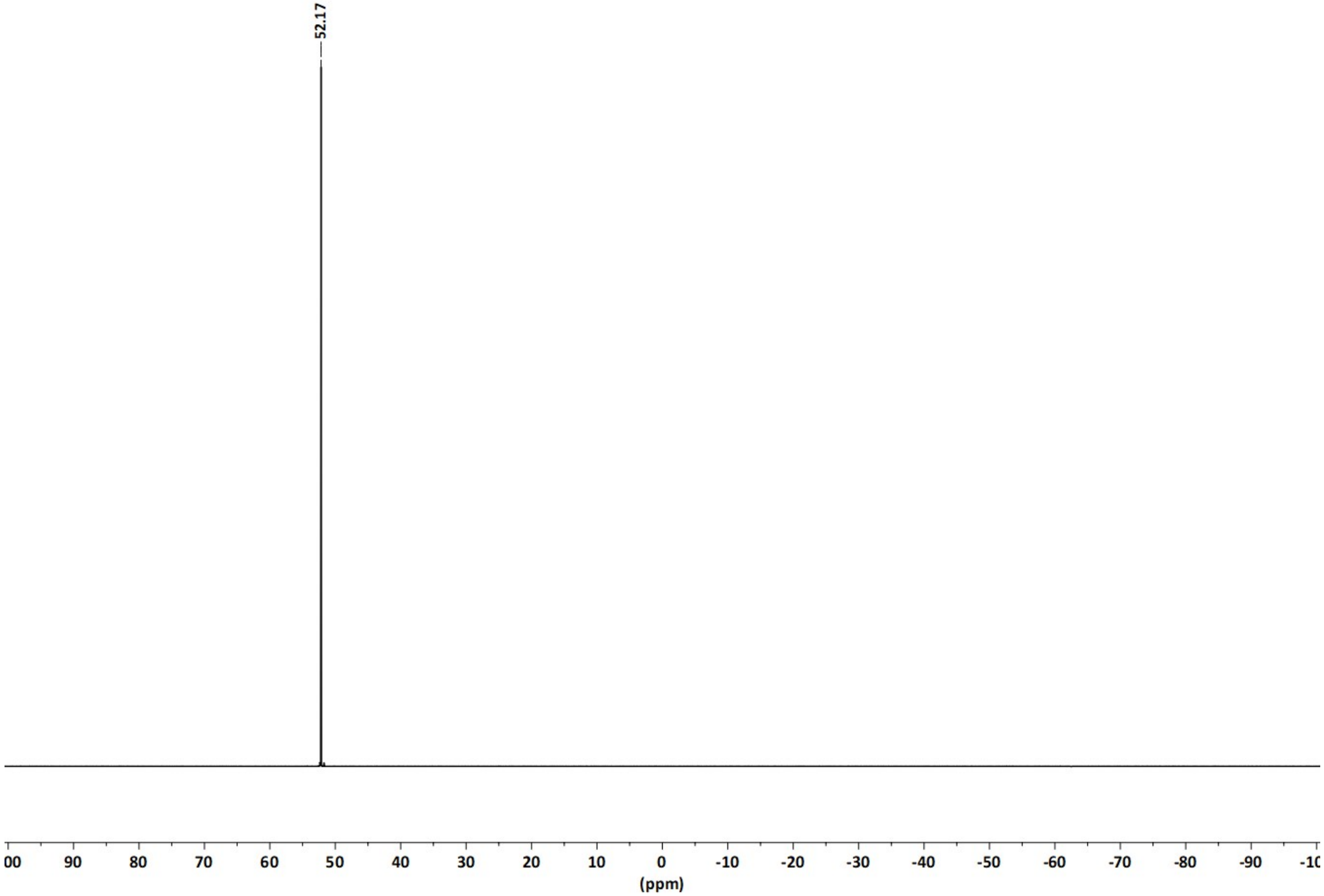

Figure S3. $^{19}F\{^{1}H\}$ NMR ($CDCl_3$, 565 MHz) spectrum of 2,6-$Mes_2C_6H_3C(O)F$

Synthesis and characterization of 2. 1 (72.0 mg, 0.20 mmol, 1.00 eq.) and $AlCl_3$ (26.6 mg, 0.20 mmol, 1.00 eq.) are placed in a Schlenk tube. To this, CH_2Cl_2 (4 mL) is added. During the addition the solution darkens to a dark yellow. After stirring for 2 hours at 20 °C the solution is layered with *n*-hexane (20 mL). After complete diffusion the solvent is removed by decantation and the remaining solid is washed with *n*-hexane (3 x 10 mL) and dried under reduced pressure to yield **2** as deep-brown crystalline solid (99.3 mg, 91 %).

^{1}H NMR (600 MHz, CD_2Cl_2): δ = 9.71 (s, br, 1H, H26), 7.65 (t, $^3J(^1H-^1H)$ = 7.66 Hz, 1H, H4), 7.42 (d, $^3J(^1H-^1H)$ = 7.52 Hz, 1H, H5), 7.17 (s, 2H, H19 and H21), 7.14 (s, 1H, H11), 6.92 (d, $^3J(^1H-^1H)$ = 7.77 Hz, 1H, H3), 2.43 (s, 3H, H14), 2.40 (s, 3H, H24), 2.36 (s, 3H, H13), 2.19 (s, 3H, H15), 2.11 (s, 6H, H23 and H25) ppm. **$^{13}C\{^1H\}$ NMR (151 MHz, CD_2Cl_2)**: δ = 200.61 (s, C16), 147.16 (s, C8), 146.98 (s, C2), 146.74 (s, C11), 145.68 (s, C6), 142.18 (s, C4), 141.78 (s, C7), 141.54 (s, C9), 140.93 (s, C20), 135.88 (s, C18 and C22), 135.22 (s, C12), 131.62 (s, C3), 130.34 (s, C19 and C21), 129.37 (s, C17), 128.99 (s C10), 128.17 (s, C1), 124.76 (s, C5), 20.97 (s, C24), 20.02 (s, C23 and C25), 19.27 (s, C14), 18.73 (s, C15), 15.44 (s, C13) ppm. **HRMS ESI (m/z):** $[M]^+$ calculated. for $C_{25}H_{25}O$ 341.18999; found, 341.18981. **UV-Vis:** (50µM, **CH_2Cl_2**) = 421 nm.

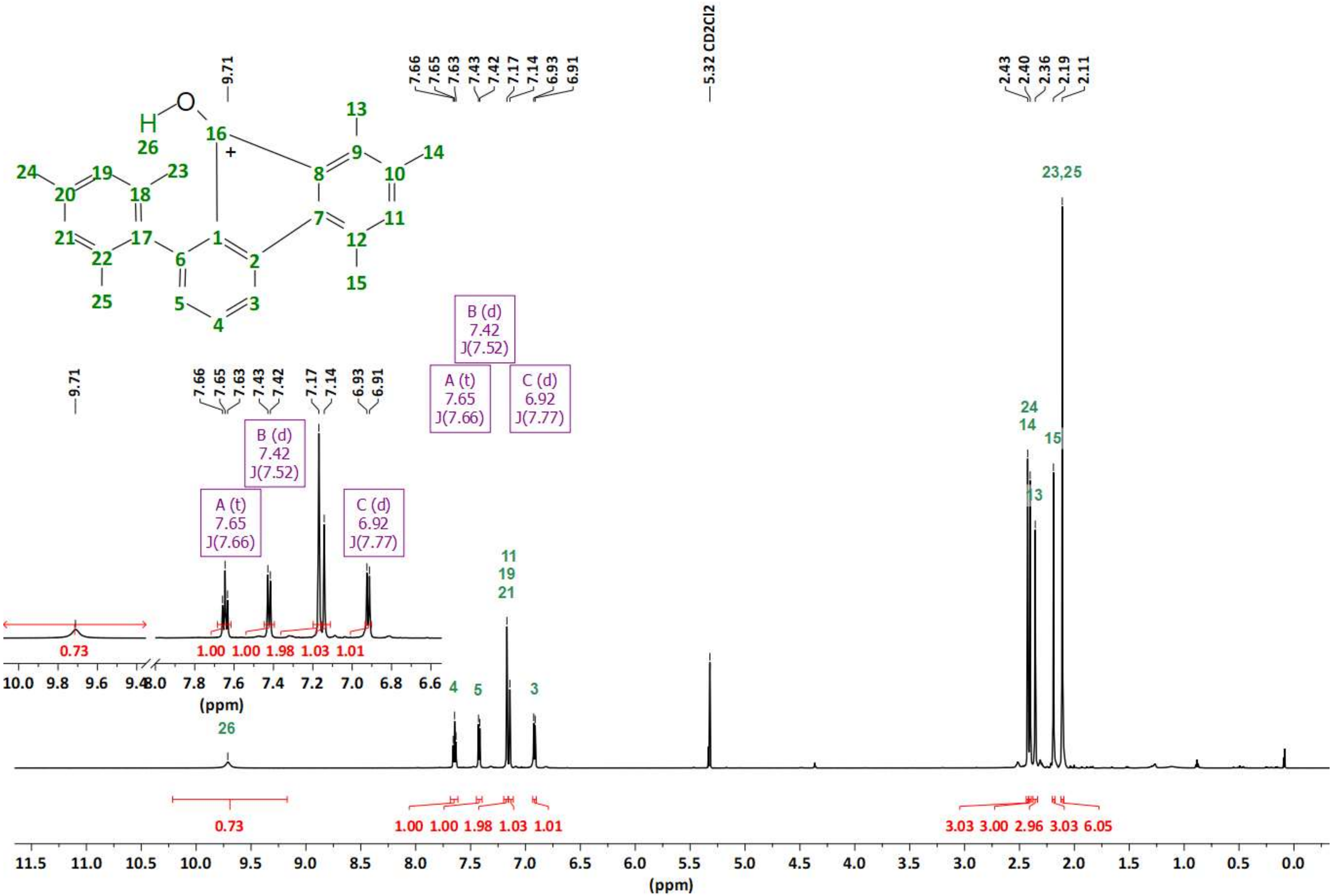

Figure S4. ^{1}H NMR (CD_2Cl_2, 600 MHz) spectrum of **1**.

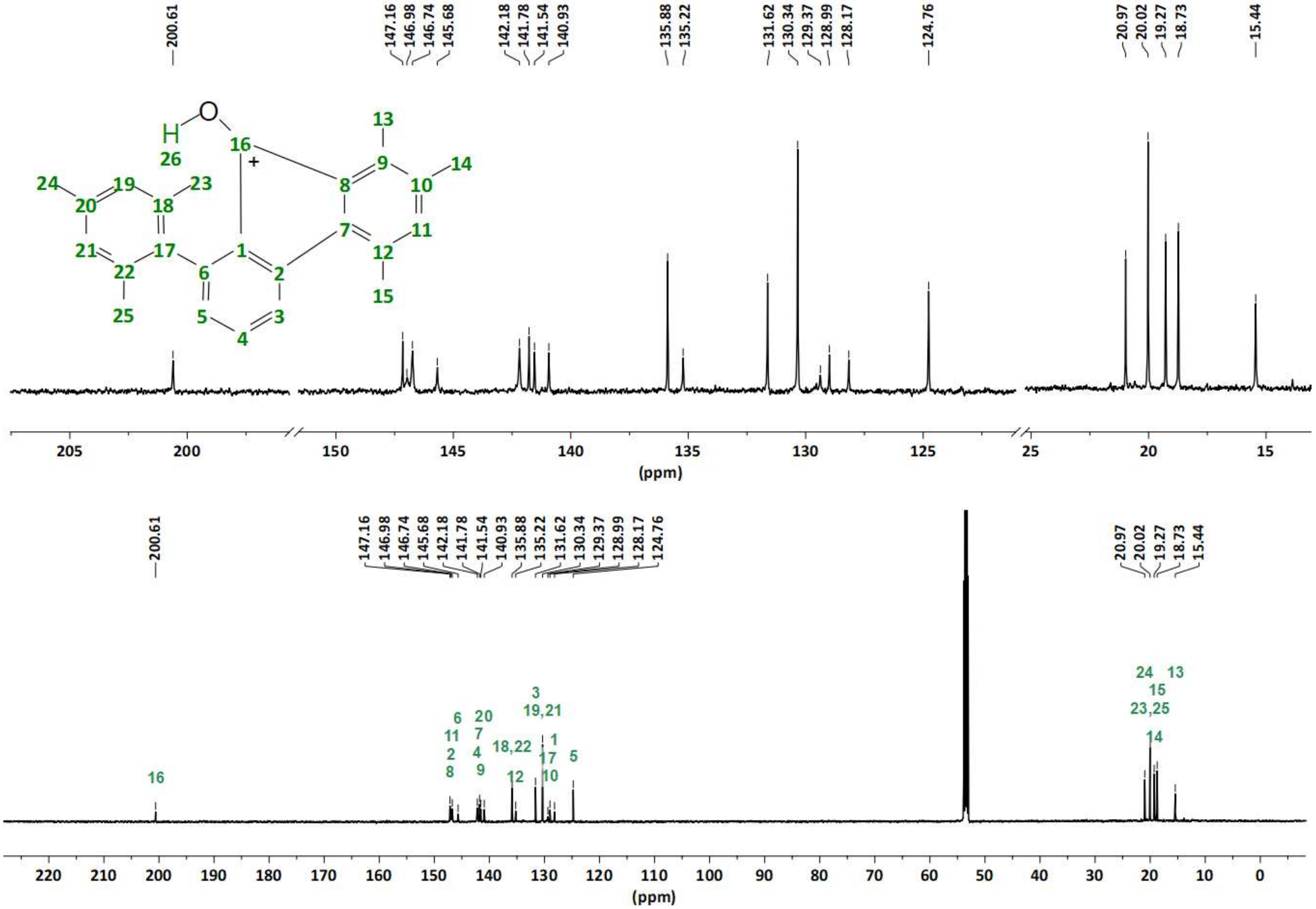

Figure S5. $^{13}C\{^{1}H\}$ NMR (CD_2Cl_2, 151 MHz) spectrum of **1**.

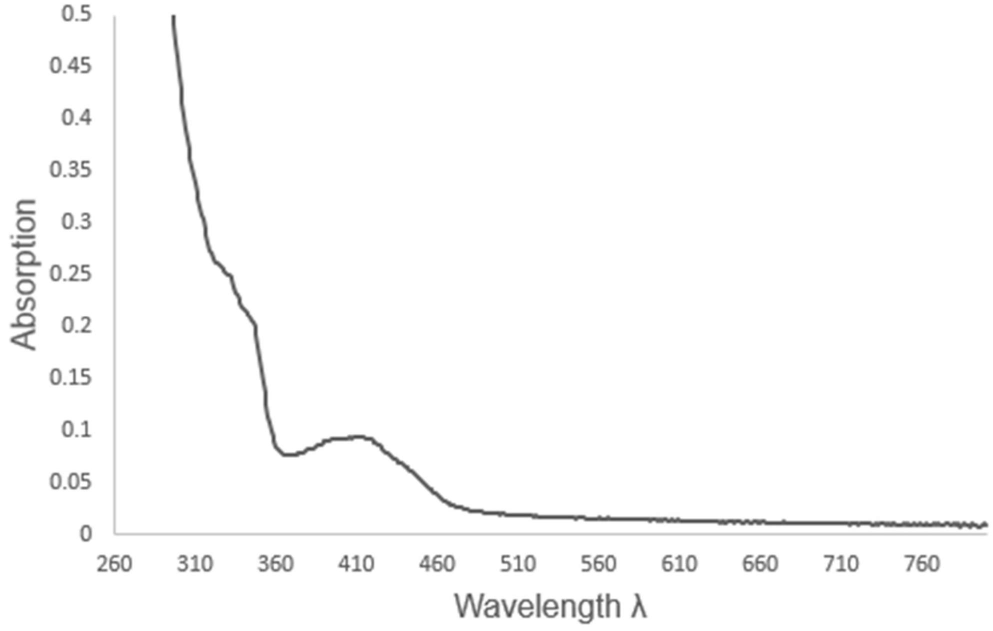

Figure S6. UV-Vis spectrum of **1** (50μM, DCM).

Synthesis and characterization of 3. A solution of **2** (51.0 mg, 0.10 mmol, 1.00 eq.) in CH_2Cl_2 (5 mL) is treated with sodium hydroxide solution (2.5 M) (0.5 mL) and stirred for 10 minutes. Afterwards the solution is filtered through a pad of Na_2SO_4. The solvent is removed in vacuum and the bright yellow oil is dissolved in *n*-hexane. The solution is flushed through a pad of silica and the product is eluted with CH_2Cl_2. After removal of the solvent in vacuum, **3** was obtained as yellow solid (34.0 mg, quant.; Mp. 176°C).

^{1}H NMR (600 MHz, $CDCl_3$): δ = 7.63 (d, $^3J(^1H-^1H)$ = 7.55 Hz, 1H, H5), 7.51 (t, $^3J(^1H-^1H)$ = 7.60 Hz, 1H, H4), 7.04 (s, H11), 7.00 (s, 2H, H19 and H21), 6.97 (d, $^3J(^1H-^1H)$ = 7.60 Hz, 1H, H3), 2.59 (s, 3H, H15), 2.49 (s, 3H, H13), 2.39 (s, 3H, H24), 2.26 (s, 3H, H14), 2.00 (s, 6H, H23 and H25) ppm. 2.34 (s, 6H, H14), 2.03 (s, 12H, H13 and H15) ppm. **$^{13}C\{^1H\}$ NMR (151 MHz, $CDCl_3$)**: δ = 195.29 (s, C16), 145.43 (s, C2), 140.26 (s, C6), 139.98 (s, C7), 139.09 (s, C9), 137.93 (s, C11), 136.77 (s, C10), 136.13 (s C12), 135.54 (s, C20), 135.13 (s, C18 and C22), 134.07 (s, C4), 131.52 (s, C1), 131.31 (s, C8), 130.48 (s, C3), 128.17 (s, C17, C19 and C21), 121.82 (s, C5), 21.36 (s, C24), 20.52 (s, C23 and C25), 20.37 (s, C15), 19.22 (s, C14), 13.17 (s, C13) ppm. **HRMS ESI (m/z):** $[M+Na]^+$ calculated. for $C_{25}H_{24}NaO$, 363.17194; found, 363.17170. **UV-Vis:** (50 µM, DCM) = 333, 346, 421 nm. **Fluorescence:** (50 µMol, 1:100 MeCN/H_2O) λ_{exc} = 320 nm, λ_{em} = 512 nm.

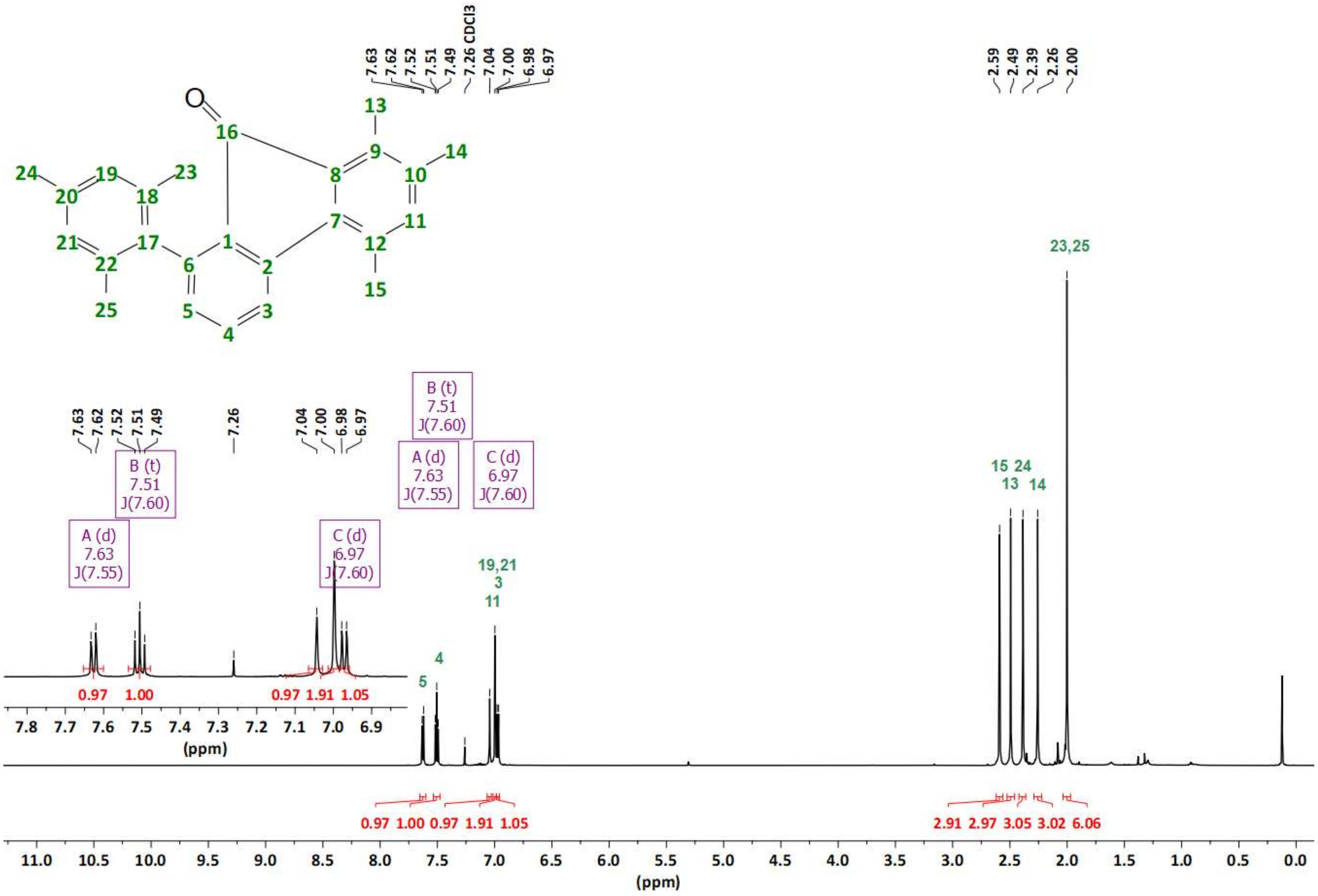

Figure S7. ^{1}H NMR ($CDCl_3$, 600 MHz) spectrum of **2**.

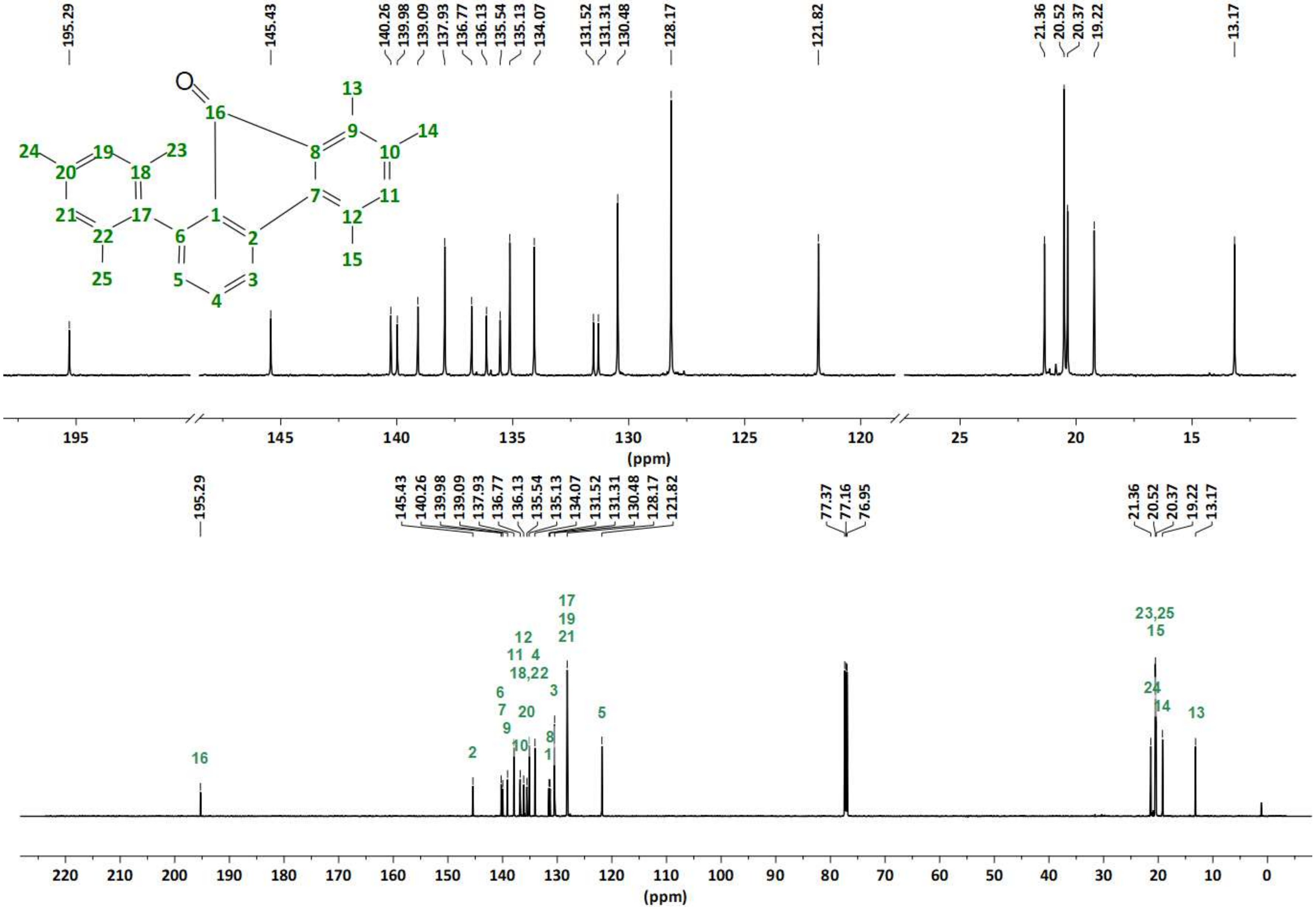

Figure S8. $^{13}C\{^1H\}$ NMR ($CDCl_3$, 151 MHz) spectrum of **2**.

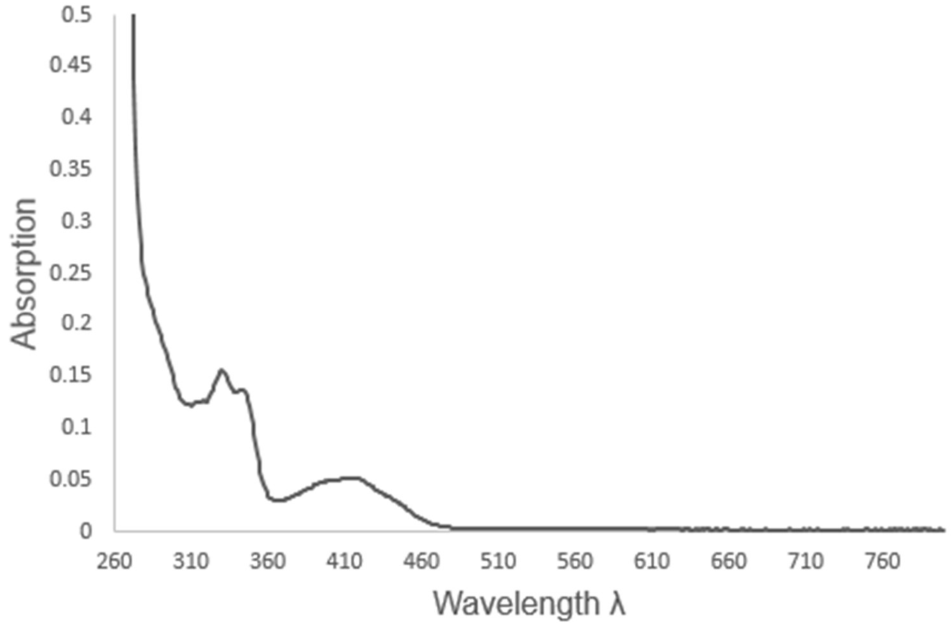

Figure S9. UV-Vis spectrum of **2** (50 μM, CH_2Cl_2).

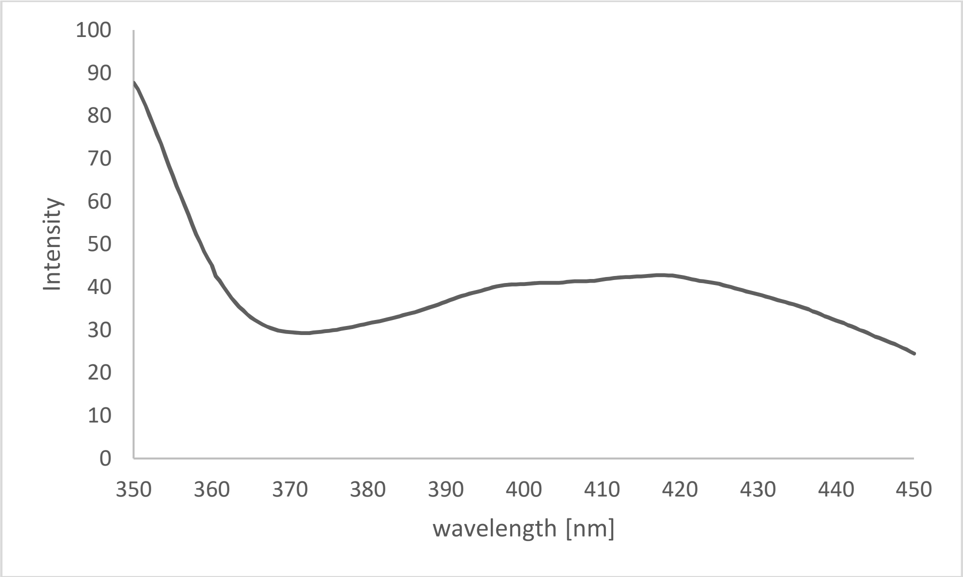

Figure S10. Excitation spectrum of **3** (50μM, 1:100 MeCN/H_2O)

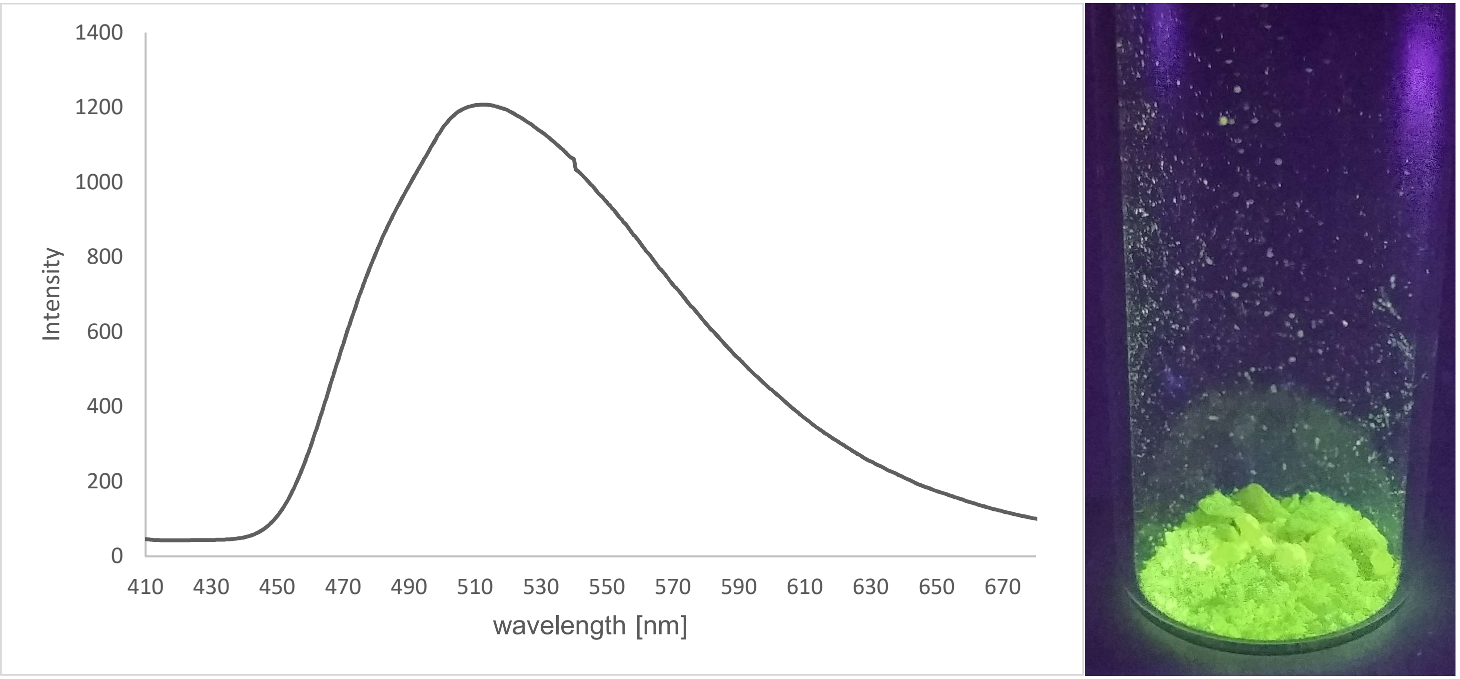

Figure S11. Emission spectrum of **3** (λ_{exi} = 320 nm, 50 μM, 1:100 MeCN/H_2O)

X-Ray diffraction studies

Single crystals of **1** and **3** were grown by slowly cooling hot solutions in toluene and *n*-heptane, respectively. Single crystals of **2** were obtained by diffusion of *n*-hexane into a CH_2Cl_2 solution. Intensity data of **1** - **3** was collected on a Bruker Venture D8 diffractometer at 100 K with graphite-monochromated Mo-Kα (0.7107 Å) radiation. All structures were solved by direct methods and refined based on F^2 by use of the SHELX program package as implemented in OLEX.[S4] All non-hydrogen atoms were refined using anisotropic displacement parameters. Hydrogen atoms attached to carbon atoms were included in geometrically calculated positions using a riding model. Crystal and refinement data are collected in Tables S1. Figures were created using DIAMOND.[S5] Crystallographic data for the structural analyses have been deposited with the Cambridge Crystallographic Data Centre. Copies of this information may be obtained free of charge from The Director, CCDC, 12 Union Road, Cambridge CB2 1EZ, UK (Fax: +44-1223-336033; e-mail: deposit@ccdc.cam.ac.uk or http://www.ccdc.cam.ac.uk).

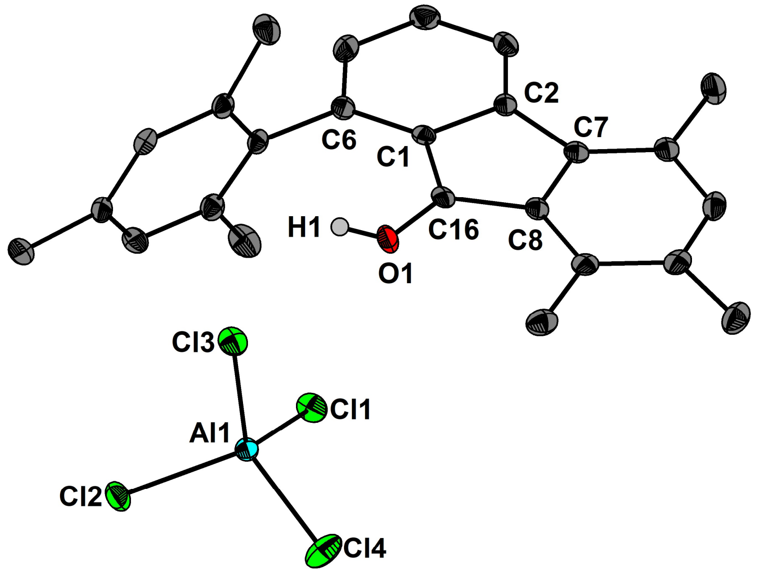

Figure S12. Molecular structure of **2** showing 50% probability ellipsoids. The closest cation anion contacts are Cl1···O1 3.234(2) Å and Cl3···O1 3.214(2) Å.

Table S1. Crystal data and structure refinement of **1** - **3**.

	**1**	**2**	**3**
Formula	$C_{25}H_{25}FO$	$C_{25}H_{25}AlCl_4O$	$C_{25}H_{24}O$
Formula weight, g mol^{-1}	360.45	510.23	340.44
Crystal system	Orthorhombic	Monoclinic	Orthorhombic
Crystal size, mm	0.29×0.28×0.17	0.14×0.13×0.11	0.25×0.23×0.17
Space group	Pbca	C2/c	$Pca2_1$
a, Å	17.4412(9)	33.2496(18)	17.6380(6)
b, Å	7.8647(4)	8.3292(5)	6.8510(2)
c, Å	28.8522(17)	18.2357(10)	31.0807(9)
α, °	90	90	90
β, °	90	104.693(2)	90
γ, °	90	90	90
V, Å^3	3957.7(4)	4885.1(5)	3755.7(2)
Z	8	8	8
ρ_{calcd}, g cm^{-3}	1.210	1.387	1.204
μ (Mo *Kα*), mm^{-1}	0.078	0.537	0.071
F(000)	1536	2112	1456
θ range, deg	2.72 to 28.28	4.654 to 56.752	4.62 to 51
Index ranges	−23 ≤ h ≤ 22<br>−10 ≤ k ≤ 10<br>−28 ≤ l ≤ 36	−43 ≤ h ≤ 43<br>−10 ≤ k ≤ 10<br>−22 ≤ l ≤ 23	−25 ≤ h ≤ 25<br>−9 ≤ k ≤ 9<br>−44 ≤ l ≤ 40
No. of reflns collected	53770	46417	61464
Completeness to θ_{max}	99.9%	99.9%	99.9%
No. indep. Reflns	4909	5634	11306
No. obsd reflns with ($I>2\sigma(I)$)	4290	4531	10082
No. refined params	250	290	481
GooF (F^2)	1.053	1.033	1.050
R_1 (F) ($I > 2\sigma(I)$)	0.0484	0.0400	0.0476
wR_2 (F^2) (all data)	0.1323	0.0892	0.1188
Largest diff peak/hole, e Å^{-3}	0.357 /−0.332	0.98/−0.78	0.30/−0.32
CCDC number	2063417	2063418	2063419

Computational data

The structures of all starting materials, transition states, intermediates, and products were optimized in the gas-phase by density functional theory (DFT) at the B3PW91/6-311+G(2df,p)[S6,S7] level of theory using Gaussian09.[S8] The starting geometries were modelled at the computer with GaussView 5. Transition states were calculated with the QST3 routine in Gaussian, using the optimized onset and end structures as starting point. Dispersion effects were accounted for using the empirical Grimme correction (GD3BJ).[S9] The wavefunction files were used for a topological analysis of the electron density according to the Atoms-In-Molecules space-partitioning scheme[16] using AIM2000,[S10] whereas NCI[17] grids were computed with NCIplot (0.1 a.u. grids).[S11] Bond paths are displayed with AIM2000, NCI figures are displayed with MolIso[S12] AIM provides a bond paths motif, which resembles and exceeds the Lewis picture of chemical bonding, disclosing all types and strengths of interactions. Analyses of the reduced density gradient, $s(\mathbf{r}) = [1/2(3\pi^2)^{1/3}]|\nabla\rho|/\rho^{4/3}$, according to the NCI method is used to visualize non-covalent bonding aspects. An estimation of different non-covalent contact types according to steric/repulsive ($\lambda_2 > 0$), van der Waals-like ($\lambda_2 \approx 0$), and attractive ($\lambda_2 < 0$) is facilitated by mapping the ED times the sign of the second eigenvalue of the Hessian ($\text{sign}(\lambda_2)\rho$) on the *iso*-surfaces of $s(\mathbf{r})$.

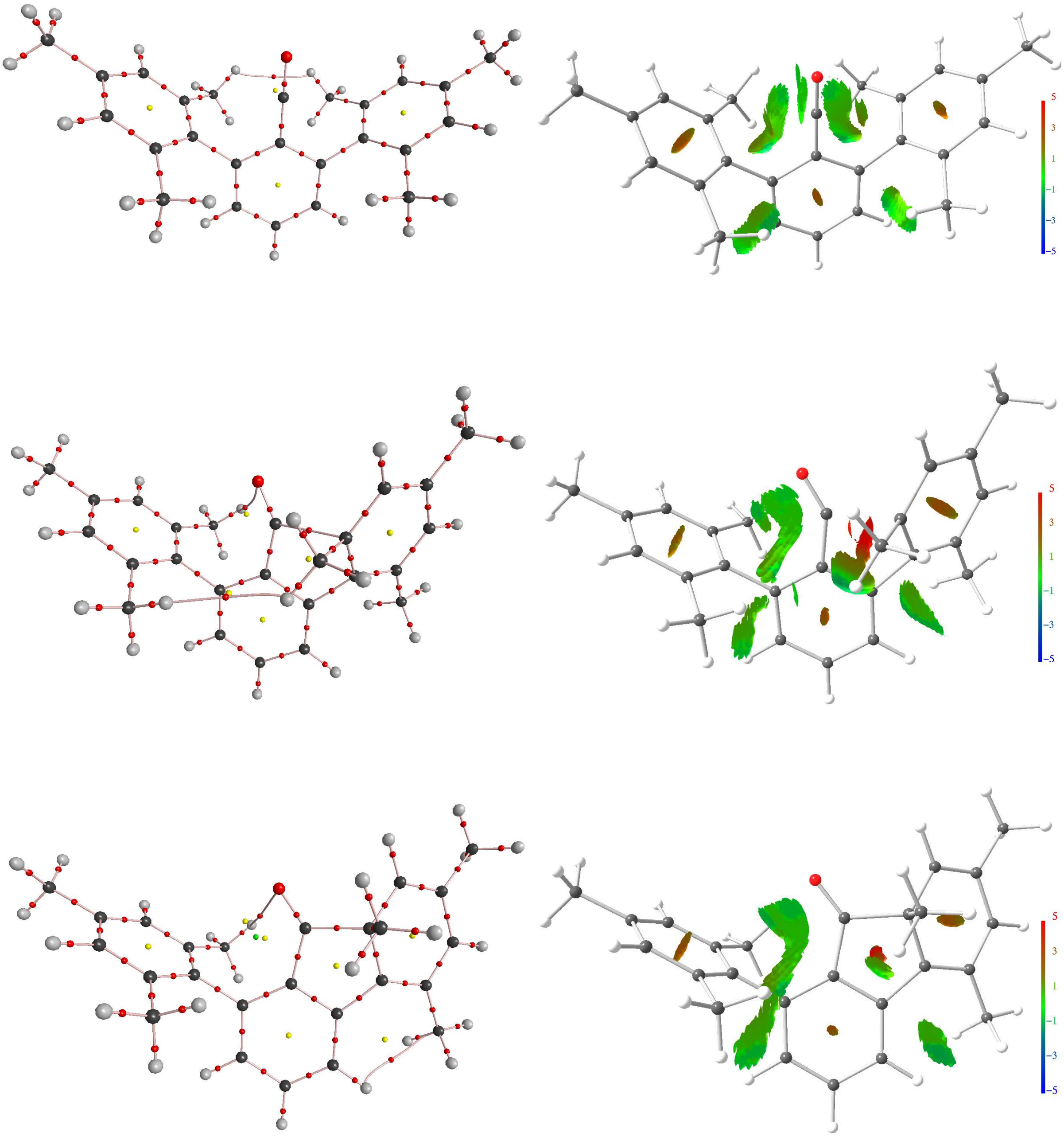

Figure S13. AIM molecular graphs and NCI *iso*-surfaces of models **A**, **B#**, and **C**.

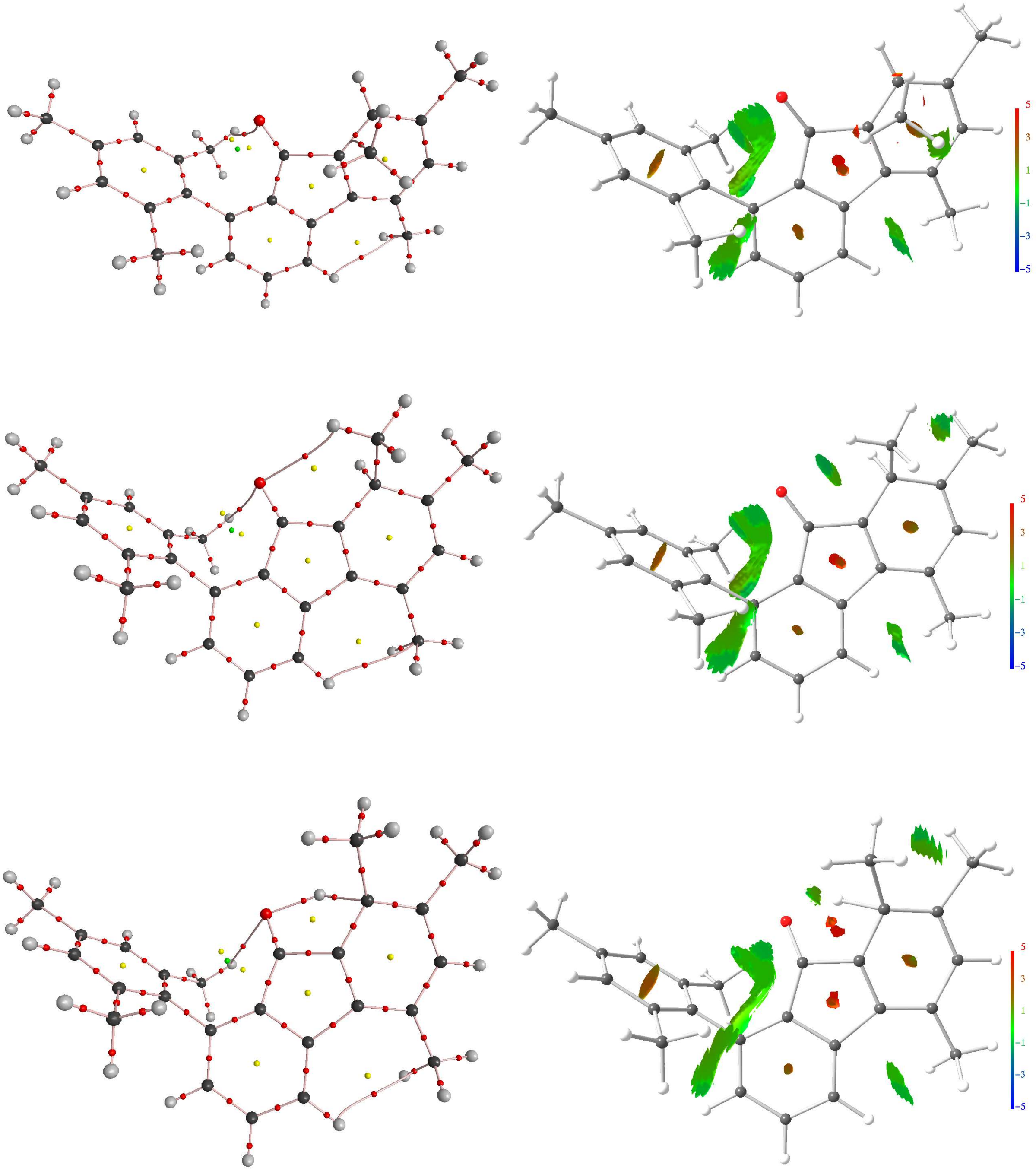

Figure S14. AIM molecular graphs and NCI *iso*-surfaces of models **D**$^{\#}$, **E**, and **F**$^{\#}$.

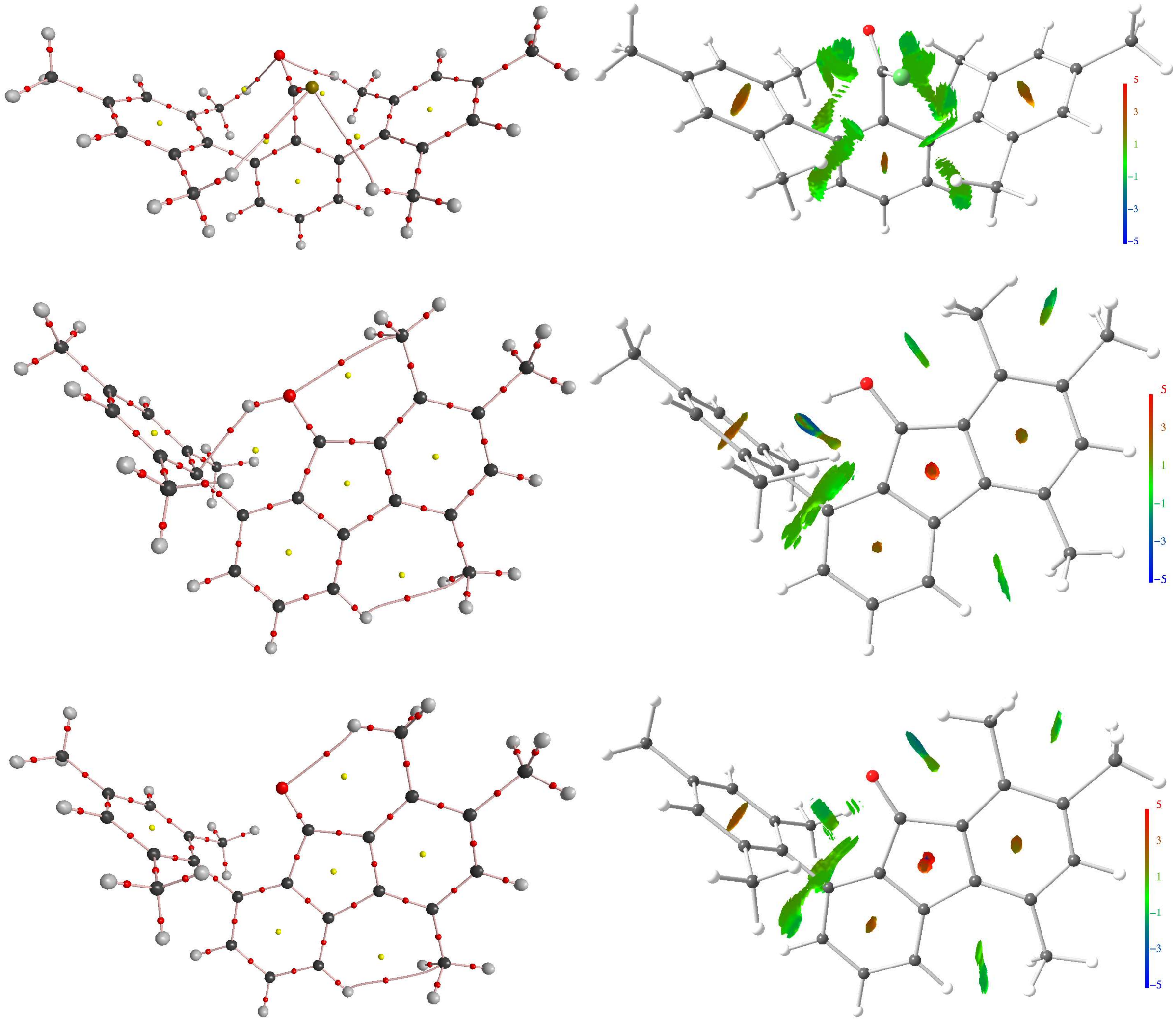

Figure S15. AIM molecular graphs and NCI *iso*-surfaces of models **1–3**.

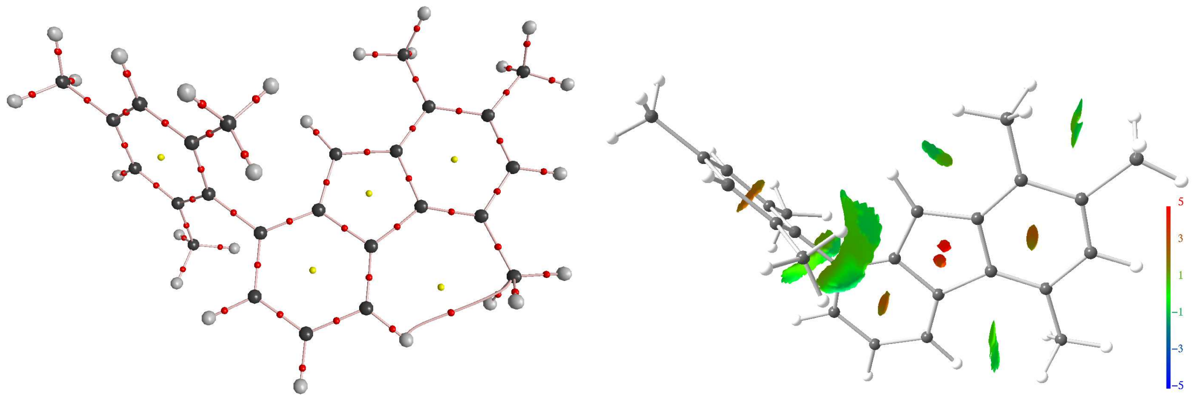

Figure S16. AIM molecular graph and NCI *iso*-surface of the non-substituted reference fluorenyl cation **H**.

Table S2. Calculated NICS(0) and NICS(1)-values

no.	1	3	A	B	C	D	E	F	G	H
51		-4.89								9.83
52		-7.02	-6.75	-4.49	-0.79	-3.33	1.33	-2.27	1.75	5.04
53	-7.36	-7.02	-8.65	-7.76	-4.60	-6.05	-3.95	-5.60	-1.61	5.03
54	-9.09	11.58	-8.47	-7.00	-4.33	-6.62	-3.75	-4.23	-1.61	24.36
55	-8.96	4.05	-5.60	-1.73	3.43	9.34	12.19	10.73	15.57	15.48
56	-7.83	4.05	-7.03	-3.81	-2.50	1.31	3.50	3.37	9.60	15.71
57	-8.80	-4.54	-7.10	-1.52	-0.79	2.59	3.76	6.36	9.60	9.89
58	-8.95	-6.70	-6.76	-7.46	-5.86	-4.43	-3.63	-2.62	0.72	5.09
59	-7.31	-6.70	-8.65	-9.32	-8.63	-6.95	-5.95	-5.18	-2.38	5.11
60	-8.97	-7.37	-8.47	-8.41	-7.77	-6.80	-5.89	-4.76	-2.38	-5.42
61	-8.93	-8.70		-7.29	-7.01	-7.18	-6.46	-7.49	-6.95	-7.47
62		-9.01		-8.98	-8.67	-8.72	-8.13	-9.17	-8.67	-7.63
63				-8.81	-8.62	-8.70	-8.13	-8.97	-8.68	

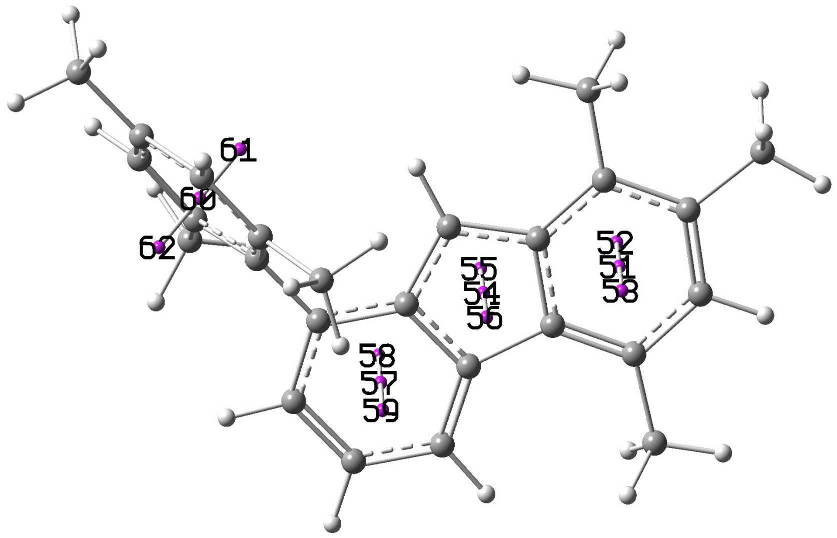

Figure S17. NICS(0) and NICS(1) points of the non-substituted reference fluorenyl cation **H**.

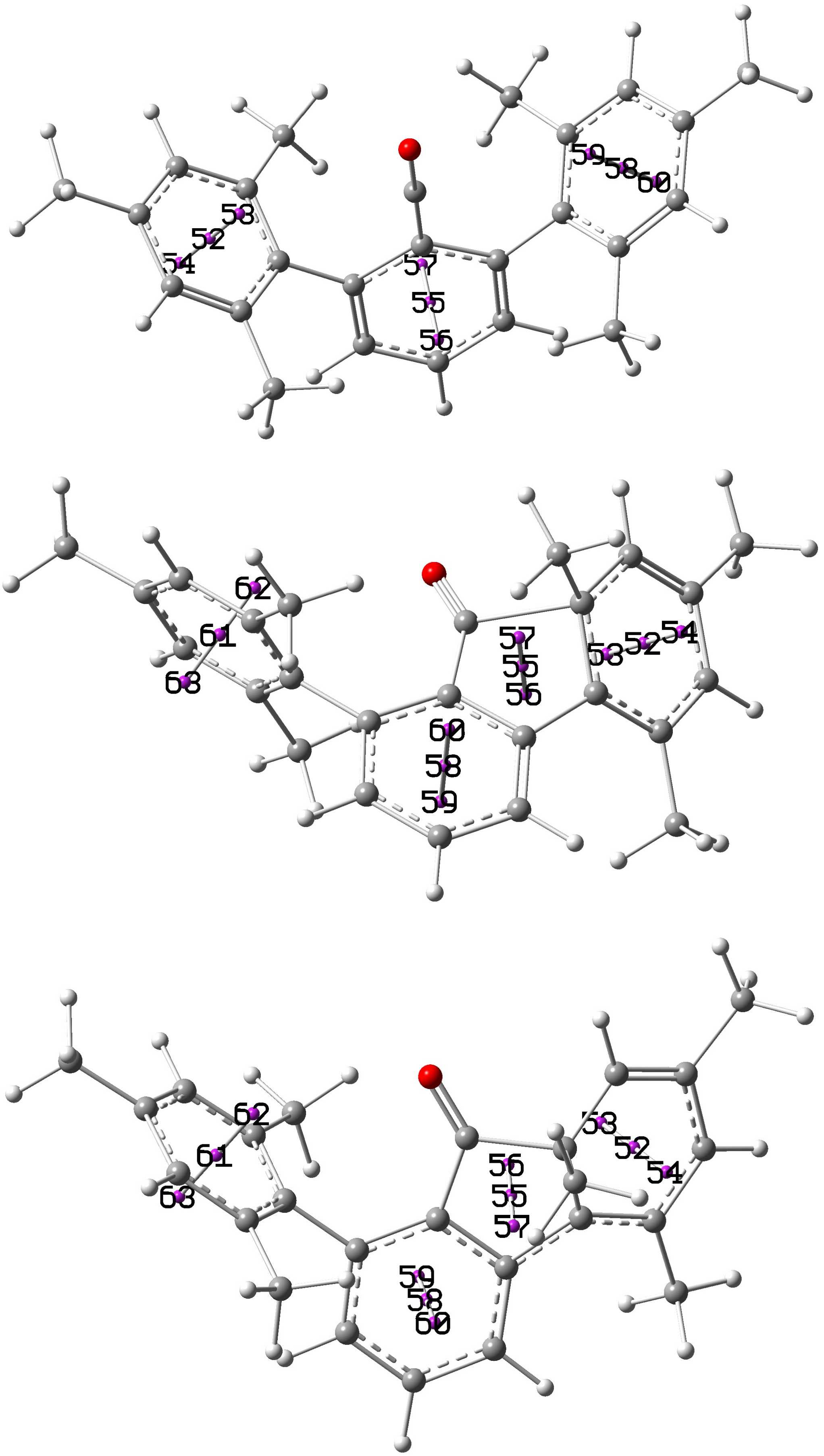

Figure S18. NICS(0) and NICS(1) points of models **A**, **B#**, and **C**.

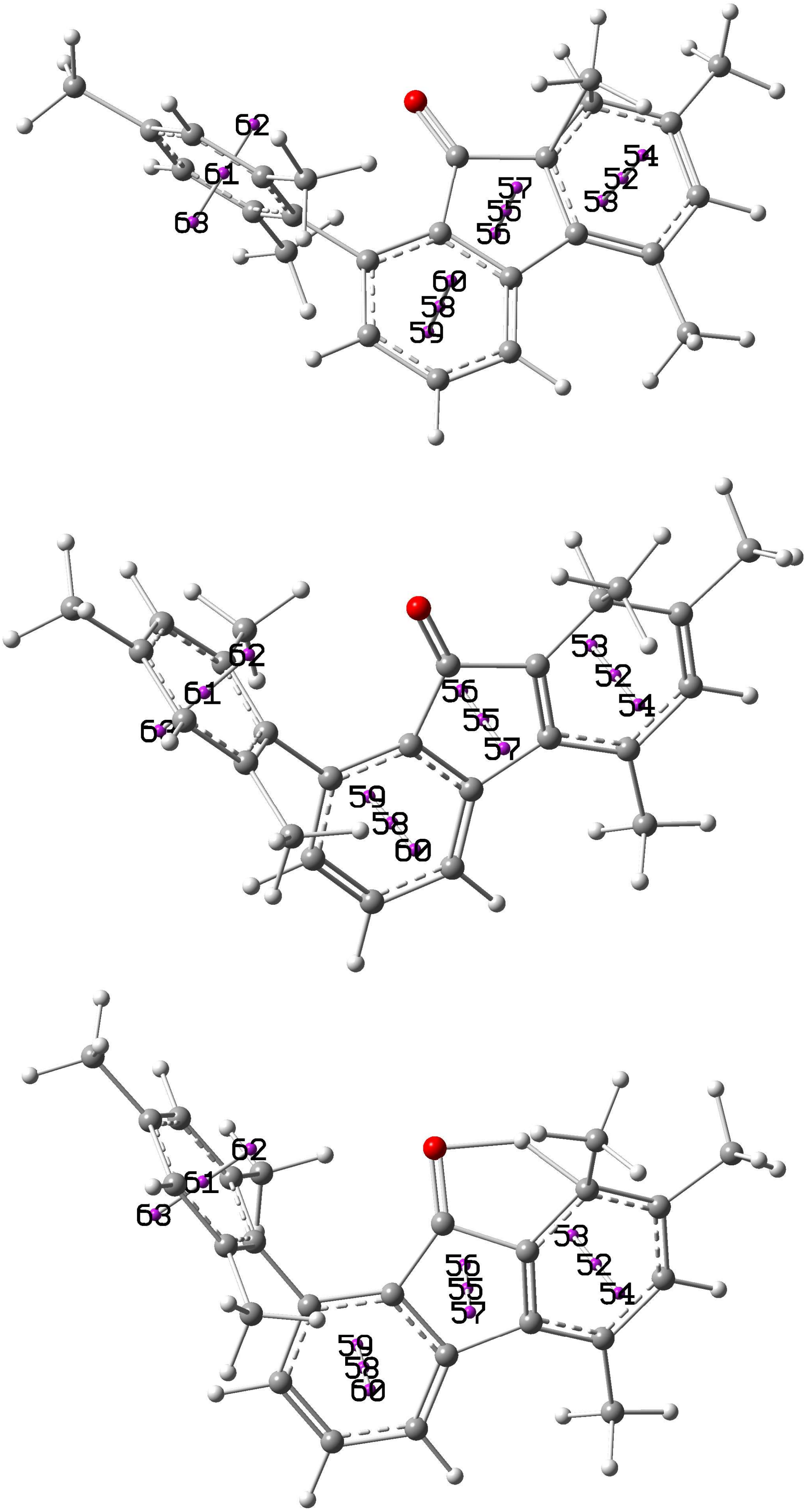

Figure S19. NICS(0) and NICS(1) points of models **D**$^{\#}$, **E**, and **F**$^{\#}$.

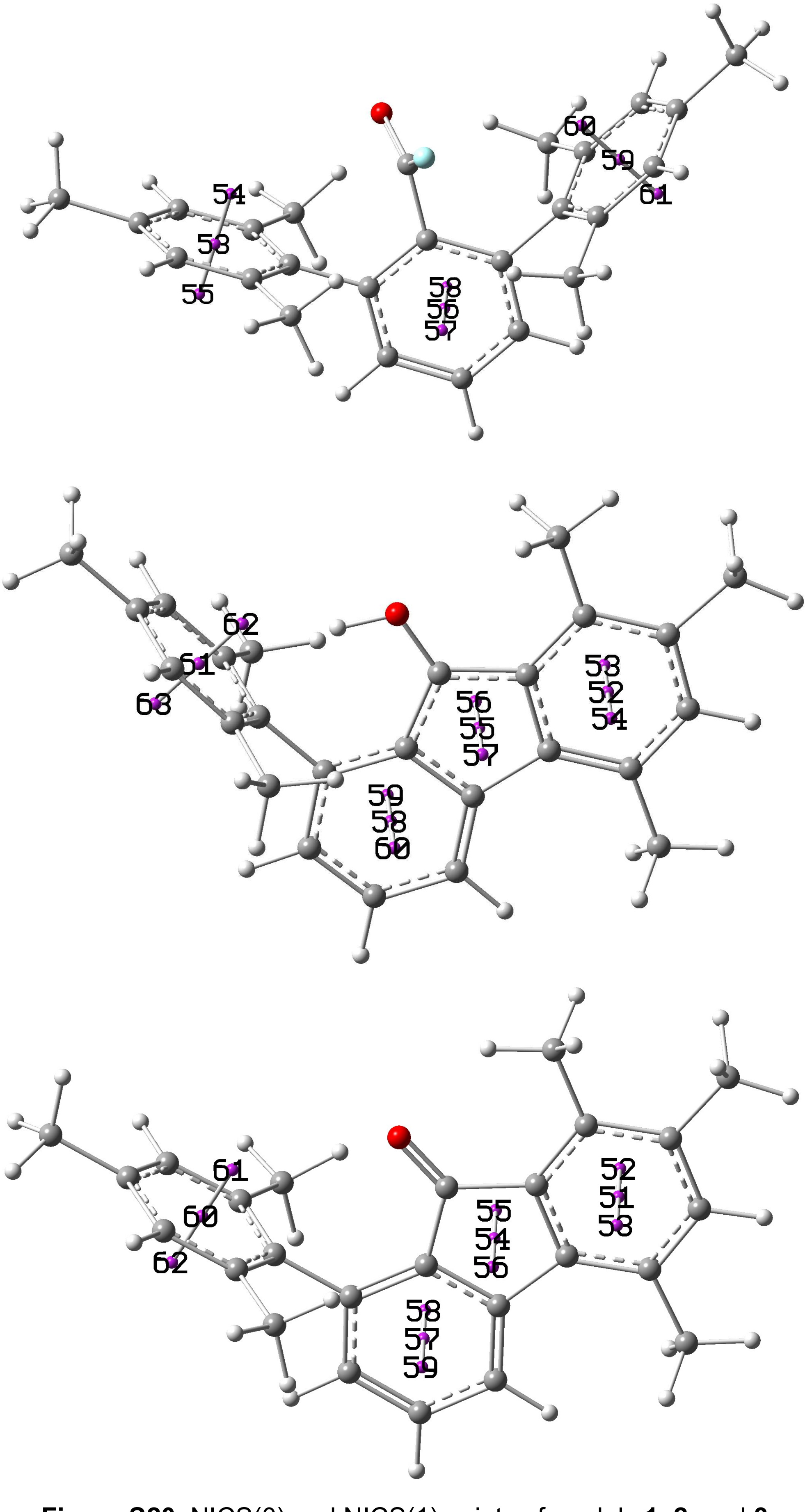

Figure S20. NICS(0) and NICS(1) points of models **1**, **2**, and **3**.

Additional References

S1. K. Ruhlandt-Senge, J. J. Ellison, R. J. Wehmschulte, F. Pauer, P. P. Power, *J. Am. Chem. Soc.* **1993**, *115*, 11353-11357.

S2. J. R. Hagadorn, L. Que, W. B. Tolman, *J. Am. Chem. Soc.* **1998**, *120*, 13531–13532]

S3. E. S. Akturk, S. J. Scappaticci, R. N. Seals, M. P. Marshak, *Inorg. Chem*. **2017**, *56*, 11466–11469.

S4. O. V. Dolomanov, L. J. Bourhis, R. J. Gildea, J. A. K. Howard, H. Puschmann, *J. Appl. Cryst.* **2009**, *42*, 339-341.

S5. K. Brandenburg, Diamond, version 4.0.4, Crystal Impact GbR: Bonn, Germany, **2012**.

S6. J. P Perdew, J. A. Chevary, S. H. Vosko, K. A. Jackson, M. R. Pederson, D. J. Singh, C. Fiolhais, *Phys. Rev*. B **1992**, *46*, 6671-6687.

S7. A. D. Becke, *J. Chem. Phys*. **1993**, *98*, 5648-5652.

S8. M. J. Frisch, G. W. Trucks, H. B. Schlegel, G. E. Scuseria, M. A. Robb, J. R. Cheeseman, G. Scalmani, V. Barone, B. Mennucci, G. A. Petersson, H. Nakatsuji, M. Caricato, X. Li, H. P. Hratchian, A. F. Izmaylov, J. Bloino, G. Zheng, J. L. Sonnenberg, M. Hada, M. Ehara, K. Toyota, R. Fukuda, J. Hasegawa, M. Ishida, T. Nakajima, Y. Honda, O. Kitao, H. Nakai, T. Vreven, J. A. Montgomery, Jr., J. E. Peralta, F. Ogliaro, M. Bearpark, J. J. Heyd, E. Brothers, K. N. Kudin, V. N. Staroverov, R. Kobayashi, J. Normand, K. Raghavachari, A. Rendell, J. C. Burant, S. S. Iyengar, J. Tomasi, M. Cossi, N. Rega, J. M. Millam, M. Klene, J. E. Knox, J. B. Cross, V. Bakken, C. Adamo, J. Jaramillo, R. Gomperts, R. E. Stratmann, O. Yazyev, A. J. Austin, R. Cammi, C. Pomelli, J. W. Ochterski, R. L. Martin, K. Morokuma, V. G. Zakrzewski, G. A. Voth, P. Salvador, J. J. Dannenberg, S. Dapprich, A. D. Daniels, Ö. Farkas, J. B. Foresman, J. V. Ortiz, J. Cioslowski, D. J. Fox, Gaussian 09, Revision B.01, Gaussian Inc., Wallingford CT, 2010.

S9. S. Grimme, J. Antony, S. Ehrlich and H. Krieg, *J. Chem. Phys*., **2010**, *132*, 154104.

S10. F. Biegler-König, J. Schönbohm, D. Bayles *J. Comput. Chem*. **2001**, *22*, 545–559.

S11. J. Contreras-García, E. Johnson, S. Keinan, R. Chaudret, J.-P. Piquemal, D. Beratan, W. Yang, *J. Chem. Theor. Comp*. **2011**, *7*, 625–632.

S12. C. B. Hübschle, P Luger *J. Appl. Crystallogr*. **2006**, *39*, 901–904.
